# Present Taste in Decoration

**Published:** 1885-10

**Authors:** 


### Present Taste in Decoration.

The general abandonment of wainscoting is all in favor of a more effective display of furniture. The beautifully patterned styles of wall paper now supplied ordinarily in subdued tones and neutral tints, with no one color unduly predominating, make, when judiciously chosen, a more effective background than one partly of wood and partly of paper. This and the fact that wainscoting is a sort of walling up, has driven it out.

The selection of the tone and pattern of paper may be properly made with reference to the intended hue and tone of the furniture and of the carpet. No arbitrary rules can be laid down in this matter. A general harmony, however, is quite consistent with strong contrasts, only these contrasts demand greater art to manage than closely analagous colors. In carpets, for instance, we now see admirable patterns in which a light field is traversed by bright and dark bands ; so also in upholstery, textile coverings of chairs, sofas, lounges, and fauteuils. The truth is that improved taste has set in against tameness ; more inspiriting effects than mere neutral colors themselves will yield are looked for. Hence the abundance of gilded and colored metal ornaments in all sorts of quaint forms, which it is now the fashion to introduce. Brilliant coloring extends even to lamp-shades, in some of which mosaic colored glass is introduced ; in others opalescent hues.—*Art and Decoration*.

